# Congenital Insensitivity to Pain Syndrome With Hidrosis in a 17-Year-Old Female

**DOI:** 10.7759/cureus.91496

**Published:** 2025-09-02

**Authors:** Robert M Paul, Amber M Parnell, Grace E Corrier, Marina Jeffery

**Affiliations:** 1 Physical Medicine and Rehabilitation, Edward Via College of Osteopathic Medicine, Monroe, USA; 2 Family Medicine, Louisiana State University Health Sciences Center, Monroe, USA

**Keywords:** autonomic involvement, charcot joint, congenital insensitivity to pain syndrome, hereditary sensory and autonomic neuropathies (hsan), hidrosis

## Abstract

Congenital insensitivity to pain syndrome (CIP) is a rare hereditary sensory and autonomic neuropathy (HSAN) characterized by impaired pain perception, recurrent injuries, chronic infections, and varying degrees of autonomic involvement. Among its subtypes, HSAN-V is distinguished by preserved cognition and sweating function but with a complete absence of pain sensation and progressive joint degeneration. We present the case of a 17-year-old female with suspected HSAN-V who developed orthopedic complications, including a chronic foot ulcer and an infected finger wound. Imaging revealed soft tissue swelling of the finger, necessitating surgical amputation, followed by a second procedure for wound closure and tendon sheath debridement. Postoperative cultures guided antibiotic therapy, and the patient recovered without complications. Genetic testing has not yet established a definitive diagnosis. This case highlights the orthopedic challenges associated with HSAN-V and underscores the importance of early recognition, multidisciplinary management, and patient-family education in optimizing outcomes and quality of life.

## Introduction

Congenital insensitivity to pain syndrome (CIP) is a rare neurological disorder in which individuals are unable to perceive pain, often leading to repeated injuries, fractures, and chronic infections. The first documented case of pain insensitivity was described by Nelaton in 1852 in a triad of brothers with plantar ulcers [1]. This early observation laid the foundation for future studies on hereditary sensory neuropathies. Later, Dyck PJ in 1975 classified these disorders into hereditary sensory and autonomic neuropathies (HSANs). This group of conditions typically presents in infancy or early childhood and is characterized by progressive sensory loss, varying degrees of autonomic dysfunction, and axonal atrophy of sensory neurons [2].

Among the five recognized HSAN subtypes, HSAN Type V (HSAN-V) is particularly rare, caused by mutations in the NGFB gene, which encodes nerve growth factor beta (NGFβ), a critical protein for the development and maintenance of nociceptive neurons [3]. Unlike HSAN-IV (congenital insensitivity to pain with anhidrosis, CIPA), which is caused by mutations in the neurotrophic tyrosine kinase receptor type 1 (NTRK1) gene, HSAN-V is characterized by a severe loss of pain perception while retaining autonomic function, including partial sweating ability [4]. This distinction is clinically important, as HSAN-IV patients experience complete anhidrosis and cognitive impairments, whereas HSAN-V patients generally have normal cognition and only partial autonomic involvement [5].

The HSAN classification system, established by Dyck PJ [6], categorizes these disorders into five main subtypes based on clinical manifestations, genetic mutations, and histopathological findings. HSAN-I presents with a late-onset sensory neuropathy with autonomic involvement, usually affecting adults. HSAN-II is an early-onset sensory neuropathy affecting both small and large fiber function. HSAN-III (familial dysautonomia) is characterized by severe autonomic instability, hypotonia, and progressive neurodegeneration. HSAN-IV (CIPA) is caused by NTRK1 mutations, presenting with complete anhidrosis and intellectual disability. HSAN-V is caused by NGFB mutations, leading to selective loss of pain perception with partial retention of autonomic function [2].

Histopathologically, HSAN-V patients show severe loss of unmyelinated nerve fibers and moderate loss of thin myelinated fibers, whereas HSAN-IV patients display reduced small myelinated fibers while retaining large myelinated fibers [5]. This differentiation is crucial for proper diagnosis and management, as the clinical and genetic overlap between HSAN subtypes can lead to diagnostic uncertainty.

Patients with HSAN-V frequently suffer from traumatic fractures, non-healing ulcers, and chronic infections due to repeated, unnoticed injuries. Without protective pain perception, minor wounds often progress to osteomyelitis and severe joint damage, leading to malunions, Charcot arthropathy, and eventual amputations [7,8]. One of the most severe orthopedic manifestations in HSAN-V is Charcot joint disease, a progressive neuropathic arthropathy caused by repeated trauma to weight-bearing joints. Over time, this leads to severe deformities, loss of joint integrity, and an increased need for surgical interventions [7].

Another complication of HSAN-V is chronic osteomyelitis, which arises from unrecognized soft tissue infections that extend to the bone. Affected individuals frequently develop painless ulcers that become secondarily infected with organisms such as methicillin-resistant Staphylococcus aureus and Streptococcus spp. [9]. These infections can progress silently until bone destruction is significant, requiring aggressive antibiotic therapy and, in severe cases, amputation. This aligns with previous reports in which osteomyelitis is a leading complication in HSAN patients [8].

In addition to orthopedic and infectious complications, oral manifestations are common in HSAN-V due to self-mutilation behaviors, particularly lip and tongue biting. Many patients require dental interventions, including primary tooth extraction, grinding of sharp edges, or the use of protective bite guards to prevent further trauma [10]. Unlike typical dental conditions where pain serves as a warning signal, HSAN-V patients often continue to damage their oral tissues unknowingly, resulting in severe ulcers and potential tooth loss [11]. 

While HSAN disorders were once considered diseases of childhood, with many patients expiring before reaching adulthood, the prognosis has improved in recent years through the use of supportive and preventive measures. However, HSAN-V remains underrepresented in the current medical literature, leading to suboptimal management and impaired quality of life [2]. Increased awareness of HSAN subtypes, their genetic underpinnings, and their multisystem complications is crucial to improving early recognition and long-term care. This case report highlights the infectious and orthopedic complications of HSAN-V in a 17-year-old female patient, emphasizing the importance of a multidisciplinary approach in managing this condition.

## Case presentation

A 17-year-old African American female presented with swelling of the left middle phalanx and an open wound on the plantar aspect of the right foot. The patient was wheelchair-bound due to multiple lower extremity injuries, including Charcot joint changes in both feet. Her mother stated that her finger had gotten caught in her wheelchair six days prior, and a decision was made to come to the hospital once the wound began draining purulent material. The foot wound was a chronic issue, currently managed by the wound care team. 

The patient’s medical history was significant for congenital insensitivity to pain syndrome with hidrosis (HSAN-V), diagnosed in infancy. She had experienced recurrent episodes of osteomyelitis, recurrent extremity ulcers, primary teeth removal secondary to self-lip biting, multiple digit amputations, and cognitive defects. Additionally, her history included numerous fractures, including a right tibial fracture, left humeral fracture, left hip fracture, and bilateral wrist fractures at various points in her life. Surgical history was significant for bilateral keratoplasty, lip surgery secondary to self-mutilation behaviors, and multiple amputations and fracture repairs. A detailed family history was limited, as the patient was adopted by her maternal aunt as an infant. Her adoptive mother was unaware of the paternal history but reported no known genetic syndromes through the maternal line. Review of systems was noncontributory, as the patient reported no pain with either of her injuries and denied all other questions asked.

**Figure 1:**
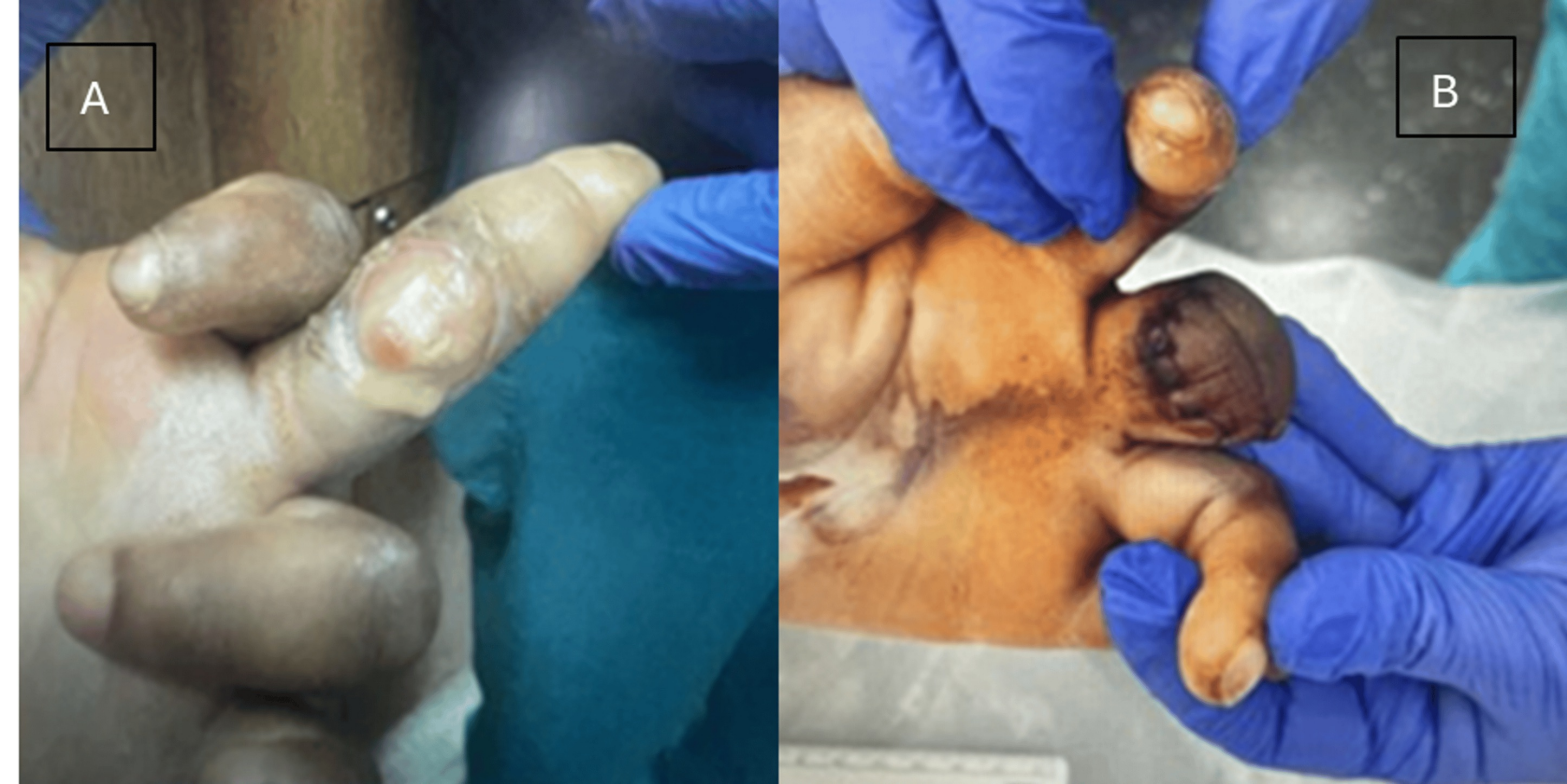
Preoperative image (A) shows a circular wound surface on the volar proximal interphalangeal joint with purulent discharge and swelling. Postoperative image (B) shows the left middle finger after amputation and tenosynovectomy, performed to control the spread of infection.

**Figure 2:**
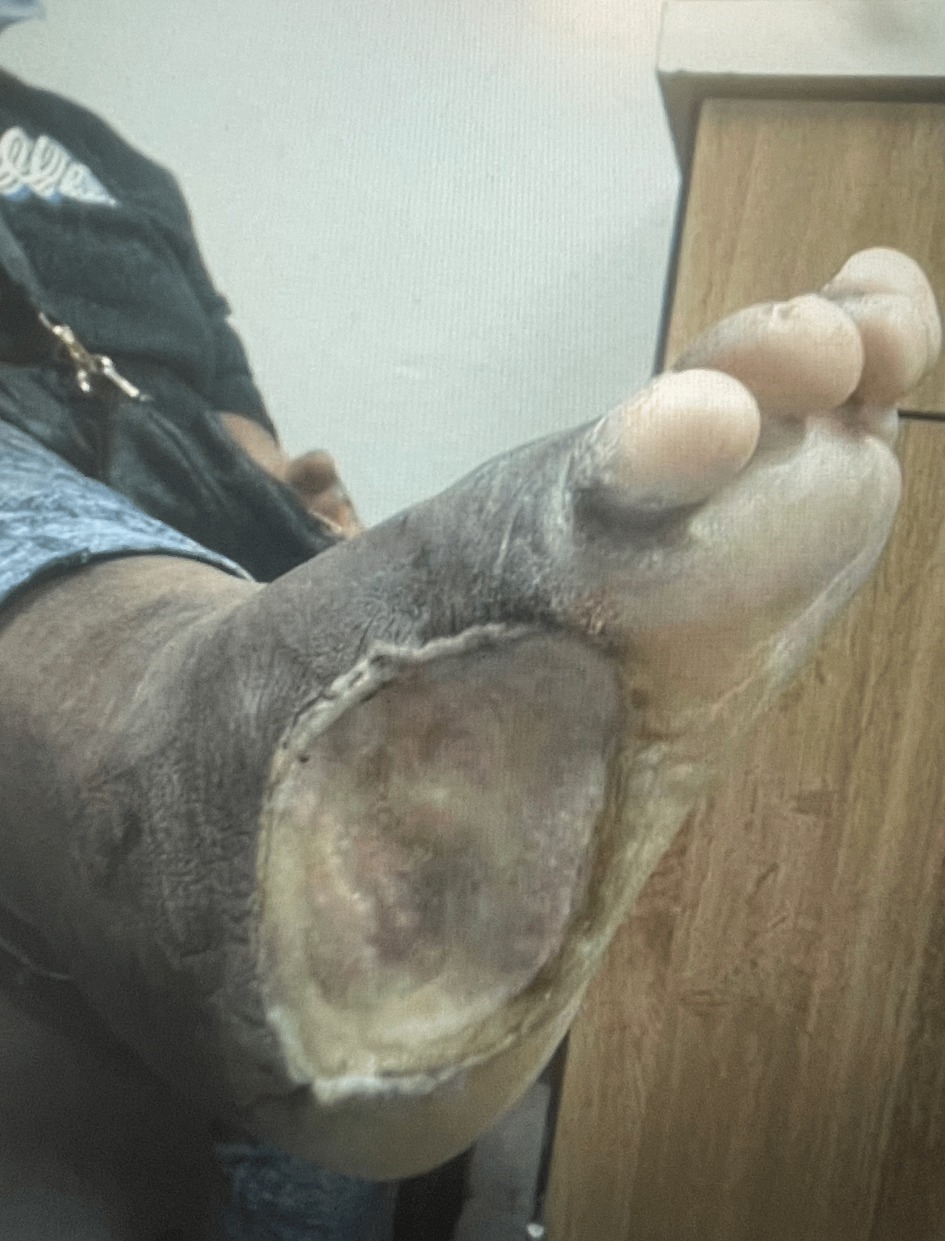
Ulceration on the plantar surface of the right foot with exposed tissue and significant swelling.

**Figure 3:**
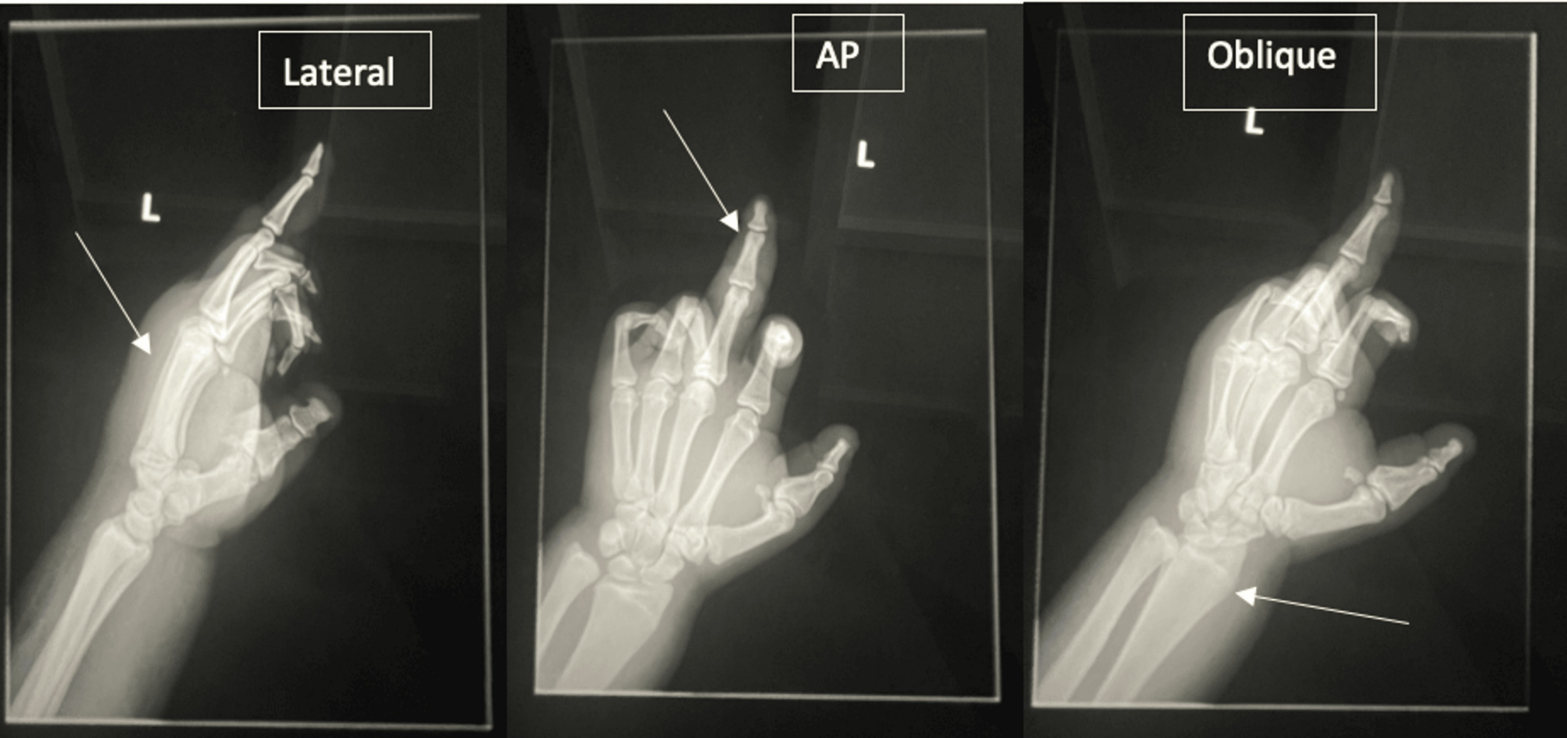
Lateral, AP, and oblique X-ray images of the patient’s left hand. No fracture or malalignment is present. Significant soft tissue swelling of the distal forearm, hand, and third digit is indicated by the arrows.

**Figure 4:**
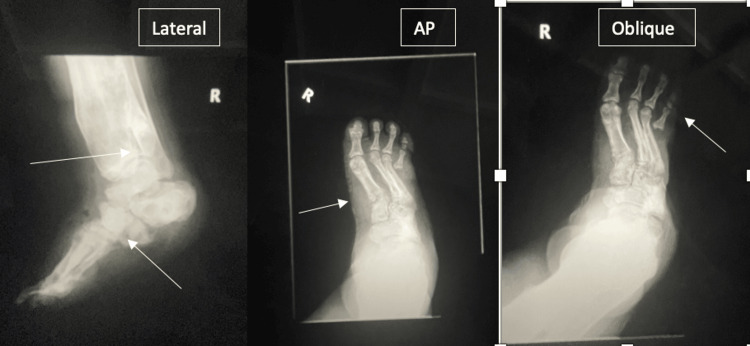
Lateral, AP, and oblique X-ray images of the patient’s right foot. In the lateral image, the top arrow indicates prior fractures of the distal tibia and fibula, while the bottom arrow highlights Charcot changes of the midfoot with gross deformity of the mid- and hindfoot. In the AP image, the arrow points to soft tissue swelling over the dorsal foot, concerning for soft tissue infection. In the oblique image, the arrow indicates bony absence of the fifth digit.

On physical examination, the left middle digit exhibited a pustular discharge (Figure 1), and the right foot exhibited an ulceration with exposed tissue and swelling (Figure 2). She was slightly febrile upon admission, though her vitals were otherwise stable. Initial laboratory findings showed leukocytosis and microcytic anemia (Table 1). Comprehensive metabolic panel revealed mild electrolyte abnormalities, including hyponatremia and hypokalemia, along with hyperglycemia, proteinemia, and hypoalbuminemia (Table 2). Radiographic imaging of the hand (Figure 3) showed soft tissue swelling of the distal forearm, hands, and fingers, particularly the third digit, consistent with cellulitis. Her chronic foot deformity was further evaluated with X-ray (Figure 4), which revealed evidence of prior fractures of the distal tibia and fibula with Charcot changes in the midfoot, along with bony absence of the fifth digit and fourth and fifth metatarsals.

**Table 1:** Complete blood count showing microcytic anemia, leukocytosis with a predominance of neutrophils (87%), and thrombocytosis.

Component	Your Value	Reference Range	Units
WBC	33.09	4.0-11.0	×10³/µL
RBC	3.48	Male: 4.7-6.1 Female: 4.2-5.4	×10⁶/µL
Hemoglobin (Hgb)	8.7	Male: 13.8-17.2 Female: 12.1-15.1	g/dL
Hematocrit (Hct)	27.3	Male: 40.7-50.3 Female: 36.1-44.3	%
Mean Corpuscular Volume (MCV)	78	80-100	fL
Mean Corpuscular Hemoglobin (MCH)	25	27-33	pg
Mean Corpuscular Hemoglobin Concentration (MCHC)	31.9	32-36	g/dL
Red Cell Distribution Width (RDW)	15.3	11.5-14.5	%
Platelets (Plt)	536	150-450	×10³/µL
Mean Platelet Volume (MPV)	8.8	7.5-11.5	fL
Neutrophils	87%	40-70%	%
Lymphocytes	9%	20-40%	%
Monocytes	4%	2-8%	%

**Table 2:** Complete metabolic profile showing hyperglycemia, hyponatremia, hypokalemia, proteinemia, and hypoalbuminemia. AST (SGOT): Aspartate Aminotransferase (Serum Glutamic-Oxaloacetic Transaminase); ALT (SGPT): Alanine Aminotransferase (Serum Glutamic-Pyruvic Transaminase).

Component	Your Value	Reference Range	Units
Glucose	171	70-99 (fasting)	mg/dL
Calcium	8.9	8.5-10.5	mg/dL
Sodium (Na⁺)	130	135-145	mmol/L
Potassium (K⁺)	3.3	3.5-5.0	mmol/L
Chloride (Cl⁻)	98	98-106	mmol/L
CO₂ (Bicarbonate, HCO₃⁻)	22	22-29	mmol/L
Blood Urea Nitrogen (BUN)	7	7-20	mg/dL
Creatinine	0.7	0.6-1.3	mg/dL
Total Protein	8.7	6.0-8.3	g/dL
Albumin	2	3.5-5.0	g/dL
Total Bilirubin	0.8	0.1-1.2	mg/dL
Alkaline Phosphatase (ALP)	82	44-147	U/L
AST (SGOT)	38	10-40	U/L
ALT (SGPT)	21	7-56	U/L

Vancomycin was started for cellulitis, and orthopedic surgery was consulted. The team opted to amputate the left middle finger (Figure 1). Postoperative tissue cultures revealed growth of methicillin-resistant *Staphylococcus aureus* (MRSA) and Streptococcus Group B. Vancomycin was continued, with ceftaroline added per the recommendation of the infectious disease team. On postoperative day 4, there was residual purulent tissue, and the orthopedic team proceeded with a secondary wound closure and tenosynovectomy. The remainder of her hospital stay was uneventful, and the patient was discharged on four weeks of sulfamethoxazole/trimethoprim (Bactrim, Sun Pharmaceutical Industries, Inc.).

During her hospitalization, the patient was evaluated by occupational and physical therapy. They noted severe limitations in her ability to perform activities of daily living (ADLs). On discharge, she was referred to an outpatient rehabilitation facility for strengthening her upper and lower extremities and improving overall functionality. Prognosis for recovery would be based on adherence to outpatient therapeutic treatments. The overall prognosis for patients with HSAN-V is a normal life expectancy, with emphasis placed on routine visits with a primary care physician to identify any pressing health risks.

No new genetic testing was performed, as she had already undergone an extensive genetic workup. Past tests included those for the NTRK1 (HSAN-IV), PRDM12 (CIP), PMP22 (CMT), and TRPV4 (dSMA) genes, all of which were negative. The patient’s adoption status has limited the availability of a comprehensive family history, which could have provided insights into the genetic basis of her condition. She is currently being managed by a pediatric neurologist with an ongoing evaluation of possible genetic abnormalities, including a whole-exome sequencing workup.

## Discussion

HSANs are rare conditions that are underrepresented in the medical literature and medical school curricula. The unique and diverse symptoms present across the HSAN subtypes pose significant challenges in proper medical management. Effective management often requires thorough workups for peripheral sensory neuropathies, rehabilitation strategies to address infections and fractures, and proper education about the syndrome for both patients and caregivers.

The patient’s presentation is consistent with classic HSAN-V features, including recurrent painless injuries, impaired wound healing, and self-reported hidrosis. Of note, she does exhibit characteristics typically ascribed to HSAN-IV, specifically corneal lesions, as well as some mild intellectual impairment. These overlapping features have been described in the literature, suggesting possible phenotypic variability within HSAN subtypes or even undiscovered genetic heterogeneity [12,13].

Although HSAN-IV is commonly linked to mutations in the NTRK1 gene and HSAN-V to mutations in NGFβ, our patient tested negative for NTRK1, while NGFβ testing has not yet been performed. Ongoing whole-exome sequencing is currently in progress to attempt to identify a potential novel or atypical mutation.

Similar diagnostic challenges have been reported in the literature, where patients with clinical features of HSAN-V lacked identifiable pathogenic mutations, emphasizing the incomplete genotype-phenotype correlation in these disorders [14].

HSAN-V primarily impacts small-diameter sensory neurons responsible for pain and temperature sensation while sparing motor function. This distinguishes it from hereditary motor and sensory neuropathies such as Charcot-Marie-Tooth disease (CMT), in which progressive motor weakness is a prominent feature. The patient’s negative genetic testing for peripheral myelin protein 22 (PMP22), associated with CMT1A, and transient receptor potential cation channel subfamily V member 4 (TRPV4), associated with distal spinal muscular atrophy, further supports a sensory-predominant neuropathy [15,16].

There is no definitive treatment for HSAN-V, making multidisciplinary management essential. Prior reports have similarly emphasized the role of multidisciplinary care, including the use of orthotics, physical therapy, and rigorous skin monitoring to reduce complications such as osteomyelitis and auto-amputation [17,18]. During her hospital course, the patient and her guardian received extensive counseling about daily skin checks, dental care, and the use of protective gear such as padded gloves and braces. Additionally, the importance of consistent follow-ups with the patient’s primary care physician was emphasized. These approaches mirror those advocated in case series and expert reviews, underscoring the value of proactive education in improving patient outcomes [19]. Long-term outcomes of the implemented management strategies have not been assessed due to the lack of follow-up at the institution where the patient was originally seen.

The patient was treated acutely in an inpatient setting and provided appropriate educational management strategies. Psychological and social aspects of living with a condition such as HSAN-V were discussed with the patient as well. At 17 years old, the patient was aware of her condition, and we reiterated the importance of routine medical care with a primary care provider. The patient does not always recognize when she has a wound or a fracture, so it is important for her adoptive mother to check daily for any ailments. Neither the patient nor her adoptive mother reported any psychosocial concerns.

In summary, this case highlights the clinical complexity of diagnosing and managing HSAN-V, particularly when phenotypic overlap and inconclusive genetic findings are present. It reinforces the need for continued research into the genetic basis and optimal care strategies for these rare neuropathies. Definitive genetic identification of the NGFβ gene is required for HSAN-V diagnosis.

## Conclusions

This case highlights the need for increased awareness of HSAN-V among clinicians. Early recognition and intervention can significantly improve patient outcomes and help prevent limb loss. Emerging research on nerve growth factor-based therapies and potential gene-targeting approaches offers hope for future treatment options. However, additional studies are needed to explore targeted therapies that address the underlying pathophysiology of this syndrome.

Further discussion of the variable symptomatology within the different HSAN subtypes is critical to assisting medical professionals in identifying this disorder, as well as in guiding definitive NGFB gene testing. Patient treatment and education may vary slightly according to presenting symptoms; however, it is imperative that a collaborative, patient-centered treatment approach be utilized to minimize the long-term morbidities associated with this condition. This case exemplifies the importance of recognizing and discussing a rare medical disorder with potentially devastating outcomes, in an effort to raise awareness among practitioners who may encounter it in the future.
